# Spatial temperature monitoring of preterm infants using a multi-modal camera setup

**DOI:** 10.1007/s11517-026-03575-3

**Published:** 2026-04-20

**Authors:** Florian Voss, Simon Lyra, Celine Noehl, Milian Brasche, Konrad Heimann, Luisa Hensel, Thorsten Orlikowsky, Steffen Leonhardt, Markus Lueken

**Affiliations:** 1 Medical Information Technology, Helmholtz Institute for Biomedical Engineering, RWTH Aachen University, 52074 Aachen, Germany; 2 Section of Neonatology, University Hospital RWTH Aachen, 52074 Aachen, Germany

**Keywords:** Deep learning, Infrared thermography, Neonatal intensive care, Temperature

## Abstract

**Abstract:**

Accurate and reliable skin temperature monitoring is critical for the thermoregulation of premature infants, but current methods using wired sensors are invasive and prone to error. Infrared thermography offers a non-invasive, wireless alternative to current wired temperature monitoring methods. This work presents a novel non-contact system for monitoring skin temperature of premature infants in closed incubators using infrared thermography. To enable temperature monitoring of specific body parts, an automated body part segmentation model was developed. The multimodal U-Net incorporated color and long-wave infrared image data. Transfer learning and data augmentation techniques improved the performance of the model, resulting in an average Intersection over Union of 0.77 across all body parts (head, torso, arms, and legs). To ensure the accuracy of the thermal data itself, a novel temperature correction algorithm was developed. This compensated for systematic errors caused by factors such as an infrared window, reflections from incubator walls, and camera drift. The algorithm achieved a mean absolute error of 0.17 $^{\circ }$C and a maximum error of 0.83 $^{\circ }$C when validated against a blackbody reference. By combining these steps, our system extracts spatial temperature using a percentile-based method, resulting in a mean absolute error of 0.41 $^{\circ }$C compared to a reference adhesive temperature sensor on the torso. The analysis revealed that the torso and arms provided more robust central and peripheral temperature measurements than the head and legs. These results demonstrate the potential of this non-contact system for accurate and reliable clinical temperature monitoring in premature infants, offering significant benefits in terms of patient comfort, reduced risk of infection and improved workflow for medical staff.

**Graphical abstract:**

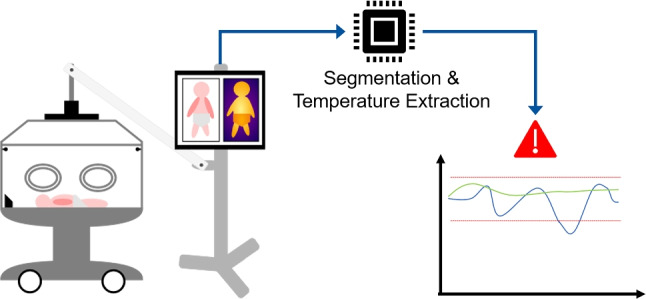

## Introduction

Globally, one in ten infants is born prematurely. This is a matter of concern since complications of preterm birth represent the leading cause of mortality among neonates [1, 2]. Infants born prematurely are at an increased risk of developing severe illnesses, both short- and long-term [3]. During the birth of the infant, their surroundings change rapidly, requiring adaptation to the new environment. One of the most significant challenges for newborns is thermoregulation, as they are unable to control their body heat and can lose heat at a rate of 0.1 $^{\circ }$C to 0.3 $^{\circ }$C per minute if they are not handled appropriately [4].

The creation of an optimal thermal environment for newborns represents a fundamental objective in the field of neonatal care. Incubators are a vital clinical tool in this regard, as they are capable of regulating the air temperature and humidity to minimize the energy expenditure of the infant and stabilize their body temperature [5].

Monitoring and regulating body temperature is of the utmost importance, as it constitutes an essential vital parameter. It is well documented that thermal stress is associated with elevated mortality and morbidity rates [6]. Furthermore, the recognition of temperature symptoms may serve as an early indicator of sepsis [7]. To guarantee continuous observation of the infant’s health condition, the temperature is commonly evaluated through the utilization of thermal skin probes. However, frequent replacements of these probes can cause skin damage and an increased risk of infection [4]. Furthermore, the use of cables can pose a source of inconvenience for medical staff and parents [8].

A contactless thermography system is a promising alternative that has the potential to become a valuable solution in neonatal care. Long-wave infrared cameras represent a promising tool for this purpose [4]. The ability to contactlessly measure multiple skin areas simultaneously could offer a more comprehensive temperature monitoring than current standards and less intrusive monitoring in general, eliminating the need for attached sensors.

However, in order to measure the temperature distribution of a premature infant with infrared thermography (IRT), certain technical requirements must be met. It is crucial to automatically segment the skin of the infant’s body parts, as the location of these regions may vary due to movement. Furthermore, measuring the temperature of premature infants requires high absolute accuracy, which infrared cameras currently do not provide. Thus, a method must be developed to guarantee robust and sufficiently precise extraction of the skin temperature.

The field of infant’s body part segmentation remains not well examined, despite recent years witnessing a notable increase in this area [9, 10].

More specifically, Asano et al. [11] created a dataset of 12 infants with 400 long-wave infrared (LWIR) images in total with a resolution of $320\times 256$ px. Head, body, arms and legs were manually annotated as ground truth. Using a standard U-Net without pre-trained weights, they achieved an accuracy of approximately 91 % and a mean Intersection over Union (IoU) of approximately 57 %. With additional effort using a Generative Adversarial Network with Self-Attention, they were able to achieve accuracies of approximately 93 % and a mean IoU (mIoU) of 70 %.

Voss et al. [12] adopted a fusion approach to the segmentation process. Here, the dataset comprised 600 color (RGB - red, green, blue) and 600 high-resolution LWIR images from 19 infants. The RGB images were manually labeled with the same four classes, as proposed by Asano et al. A U-Net architecture with two individual encoder branches was implemented for a fusion approach to the RGB and LWIR images. In addition, the network was pre-trained on the publicly available Crowd Instance-level Human Parsing (CIHP) dataset [13] and a gray version of CIHP in the case of the IRT encoder branch. The fusion model demonstrated superior results with an mIoU of 0.87. In comparison, the use of only RGB images produced a slightly inferior result, with an mIoU of 0.84, while the use of only LWIR images resulted in an mIoU of 0.75.

Moreover, different methods have been examined to improve the accuracy of temperature measurement using IRT. Abbas et al. [14] were the first to investigate the influences on IRT in a neonatal clinical setting, including convective incubators. A model-based correction was developed to improve the accuracy of the measurement. However, this study did not employ a thermal reference and did not undertake direct accuracy measurements with a blackbody in the clinical setting.

In contrast, Hamada et al. [4] evaluated the accuracy of infrared cameras placed inside an incubator. Using two uncooled LWIR cameras, a passive blackbody for reference and an active blackbody for measurement, they evaluated 24 scenarios with varying incubator and blackbody temperatures and humidity. A compensation method was developed that scaled the difference between the measured object and reference temperatures, added an offset, and incorporated the unscaled flux of the reference. The scaling and offset factors were determined by regression analysis on two selected scenarios. After outlier removal, this approach resulted in a mean absolute error (MAE) of less than 0.3 $^{\circ }$C for the high-cost FLIR A35 camera in all scenarios, while the low-cost Lepton 3.5 camera achieved this accuracy in 19 of the 24 scenarios.

The available research addressing this particular application has limitations, with studies either focusing exclusively on improving accuracy through correction algorithms [4, 14] or focusing only on automatic body part segmentation without investigating temperature measurements [10–12].

While related research exists, it often targets different populations or measurement types. Research into contactless temperature measurement for adults, for example, is a more established field, and it was intensified during the worldwide COVID-19 pandemic [15–18].

Other approaches have focused on metrics that do not require absolute temperature values, such as the central-peripheral temperature difference (cpTD). Because this metric relies on the subtraction between two body regions, any uniform measurement offset is cancelled out, removing the need for high absolute accuracy [19, 20]. However, the unique clinical environment of the neonatal incubator introduces novel complexities that differentiate our challenge from research on adults or non-absolute measurements.

To the best of our knowledge, there is no existing approach that describes a clinically validated IRT-based approach for automatically measuring the absolute temperature distribution of preterm infants with high accuracy.

This work presents a novel approach to accurately measuring the temperature distribution of preterm infants. This approach integrates infrared thermography (IRT) with a robust algorithm for automatic skin segmentation and a newly developed temperature correction algorithm to ensure high accuracy. For this purpose, the segmentation algorithm presented in [12] was adapted to a new clinical environment. In addition, a temperature correction algorithm was developed and evaluated with a precise blackbody to ensure reliable accuracy results. Finally, the clinical validity of this approach was demonstrated in a validation study.

## Materials and methods

### Clinical setup

The clinical study of this work was conducted in the neonatal intensive care unit of RWTH Aachen University Hospital (UKA), Aachen, Germany, following approval from the UKA ethics committee (EK 22-315). Prior to participation, written informed consent was obtained from the parents of all patients.

All clinical study recordings were collected with the use of a Thermocare Vita incubator, manufactured by Weyer GmbH in Germany. Usually, standard incubator hulls are intransparent to infrared radiation. As a solution, a *ClearIR-4-P* (Iriss) infrared window, which is transparent to long wavelength infrared radiation (LWIR, 8-14 µm), has been integrated into the lid of this research incubator. The incubator is equipped with two sensors for monitoring the air temperature by default.

To provide a ground-truth measurement, the standard adhesive skin probe routinely used for patient monitoring (Weyer GmbH, Germany) was attached to each infant’s torso. This sensor is an NTC thermistor (YSI 400 curve) with a measurement accuracy of $\pm 0.1^\circ \text {C}$ in the range of 25 $^{\circ }$C to 45 $^{\circ }$C

**Fig. 1:**
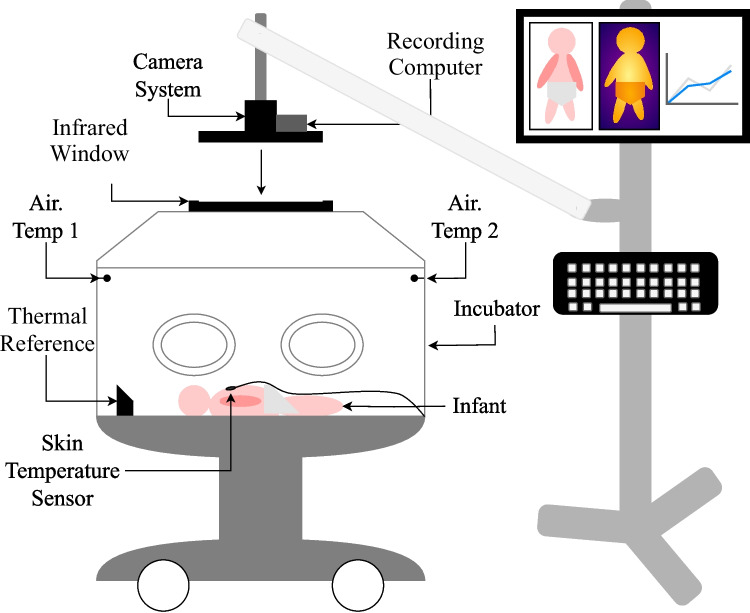
Schematic representation of the clinical hardware setup

The camera system, incorporating both RGB and infrared cameras, was placed in a central position on top of the incubator. This configuration, along with the recording computer (Jetson Xavier AGX from Nvidia), was housed within a custom-made mount, securely fixed to a medical cart. A schematic representation of the hardware setup is provided in Fig. 1.

The RGB images were acquired with a 12-megapixel OAK-D camera (Luxonis). The LWIR images were taken with a PI 640 camera from Optris. The key properties of this LWIR camera are detailed in Table 1.

**Table 1:** Properties of the used IRT camera

Property	Optris PI 640
Resolution	640 x 480
Thermal sensitivity	75 mK
Absolute accuracy	± 2 $^{\circ }$C
Field-of-view	90$^{\circ }$ x 66$^{\circ }$
Price	7500 €

In addition, a thermal reference (see Section 2.4) was placed inside the incubator to calibrate the infrared camera.

### Recordings

The dataset for this work includes 14 recordings from 14 different premature infants. In each recording, both imaging modalities were captured at one frame per second over a period of five hours. Along with the images, skin temperature measurements from the torso and all incubator temperature data were recorded at a sampling rate of 1 Hz. An overview of the patients is given in Table 2.

**Table 2:** Overview of all patients in the clinical study

ID	Gender	Gestational age (weeks)	Birth weight (kg)	Birth height (cm)	Age (days)	Record. weight (kg)	Selected Images
PAT01	m	31	1.1	38	8	1.05	50
PAT02	m	31	0.8	37	15	1.07	50
PAT03	f	30	1.13	38	11	1.035	60
PAT04	f	29	1.225	40	10	1.25	81
PAT05	f	33	1.38	39	8	1.285	99
PAT06	m	29	1.05	38	17	1.32	107
PAT07	m	29	1.09	35	14	1.23	11
PAT08	m	30	0.965	37	14	1.08	38
PAT09	f	28	0.81	34	26	1.09	96
PAT10	f	31	1.41	41	14	1.41	101
PAT11	f	31	1.39	40	14	1.52	99
PAT12	m	27	1.18	36	20	1.29	38
PAT13	f	28	0.935	35.5	22	1.26	58
PAT14	f	27	0.69	31	38	1.21	71
Mean	8 $\times$ f	29.57	1.08	37.11	16.5	1.22	68.5
	6 $\times$ m						

**Fig. 2:**
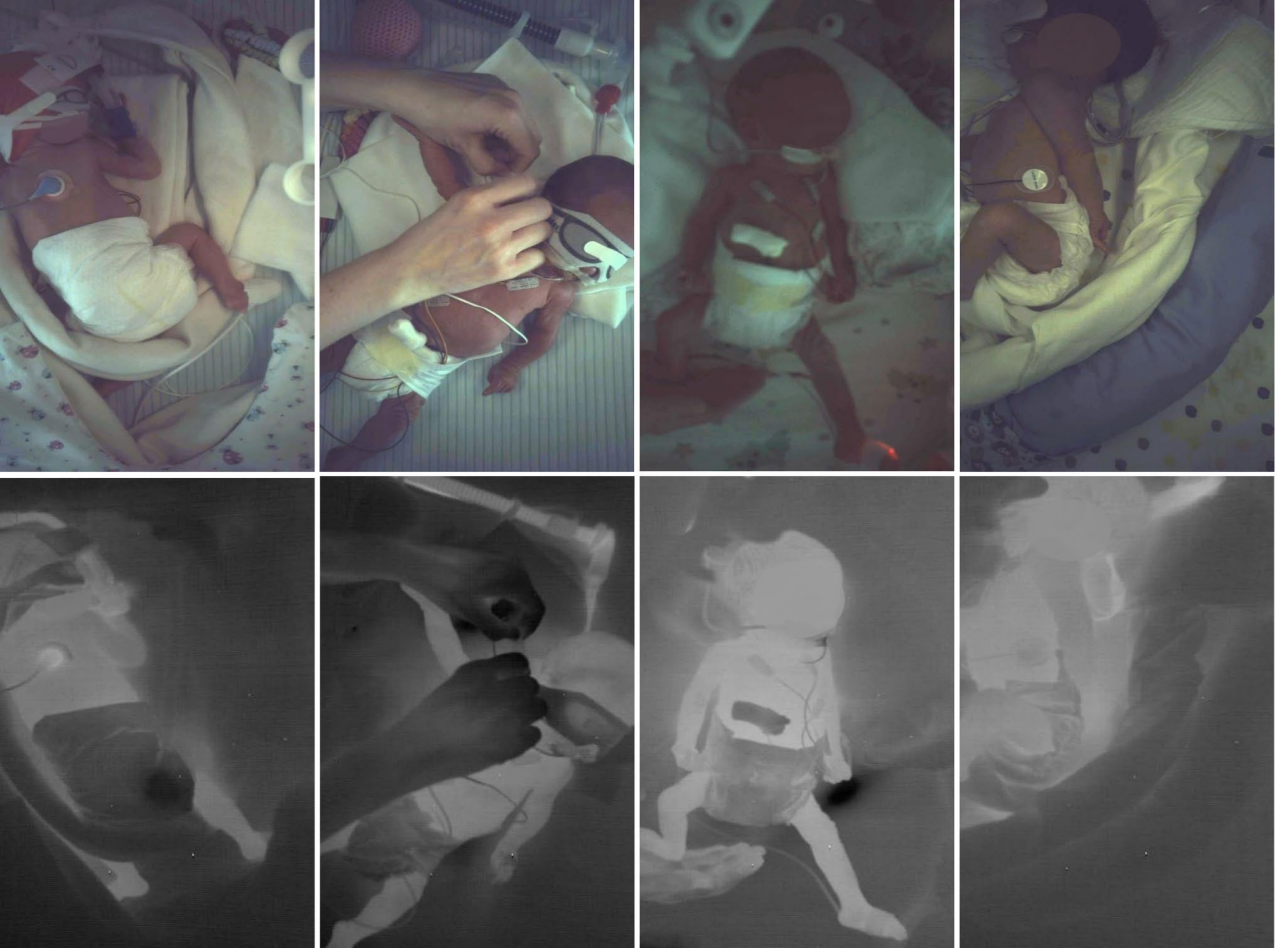
Example images of the dataset. Top: processed RGB images; Bottom: corresponding, registered LWIR images

The infants had a gestational age of 27 to 33 weeks at birth and a birth weight of 0.69 kg to1.41 kg. At the time of the recordings, they were between 8 and 40 days old and weighed between 1.05 kg to 1.52 kg. The inclusion criterion for the study was a minimum weight of 1 kg. Example images from the dataset are illustrated in Fig. 2.

### Body part segmentation

From all recordings, 959 image pairs were selected for manual pixel-wise labeling to serve as the ground truth. Since labeling the entire video dataset is unfeasible, a representative subset was chosen to ensure high diversity in posture, position, and lighting. We minimized redundancy by downsampling prolonged periods of inactivity (sleeping) and excluded frames with severe motion blur or total occlusion to ensure accurate annotation. The number of selected images per infant (11 to 107) varies depending on the duration of valid, diverse activity available in each recording.. The last column of Table 2 presents an overview of the number of selected images per patient.

After image selection, the pairs were manually labeled regarding the following categories: arms, legs, head, torso and background. Skin covered by clothing and hands or arms of caregivers were classified as background.

#### Preprocessing

Manual pre-processing was performed on both image modalities due to low brightness and a green cast in the RGB images and low contrast in the LWIR images. The green cast is due to the Bayer pattern in the RGB camera’s raw data, which interleaves red, green and blue filters, with twice as many green sensors. This pattern makes green more sensitive to noise and uneven lighting, resulting in a green cast if not corrected by white balance algorithms.

Preprocessing was performed using an automated pipeline implemented in Python with the OpenCV library. Parameters were determined empirically to standardize image quality:

- White Balance: The Gray World Assumption was applied.
- Brightening: Images with an average pixel intensity $< 70$ were brightened by a factor determined via linear interpolation, scaling from 1.5 at the threshold (70) to 3.0 at zero intensity to enhance visibility.
- CLAHE: Applied to LWIR images with a clip limit of 2 and a tile grid size of $8 \times 8$ to enhance local contrast without over-amplifying noise.

To align the LWIR and RGB images, we used the RANSAC algorithm for homography transformation due to its superior results. Six corresponding points on five pairs of images were selected, resulting in 30 pairs, which were used to estimate the homography transformation matrices. Since the camera orientation was slightly different for each recording, the registration was calculated separately for each patient. We evaluated the algorithms using RMSE for both spatial dimensions and mean absolute error. For the RANSAC algorithm, the averaged results over all recordings were RMSE$_\text {x}$ of 6.74, RMSE$_\text {y}$ of 6.24, and a mean absolute error of 7.06. We also investigated automatic feature-based registration. However, multiple approaches yielded unsatisfactory results. For this reason, a static homography transformation was chosen for image alignment.

#### Architecture

Based on the success of U-Nets in body part segmentation [12] and similar semantic segmentation tasks with image fusion [21], we chose this architecture in combination with a DenseNet121 used for feature extraction. We adopted a U-Net structure with a DenseNet-121 backbone for the encoder, incorporating skip connections to allow the transfer of high-resolution information from the encoder to the decoder. To address the specific challenges of the clinical dataset (e.g. variability in image quality, partial occlusions) and to explore the benefits of multimodal data fusion, we investigated different model configurations.

**Fig. 3:**
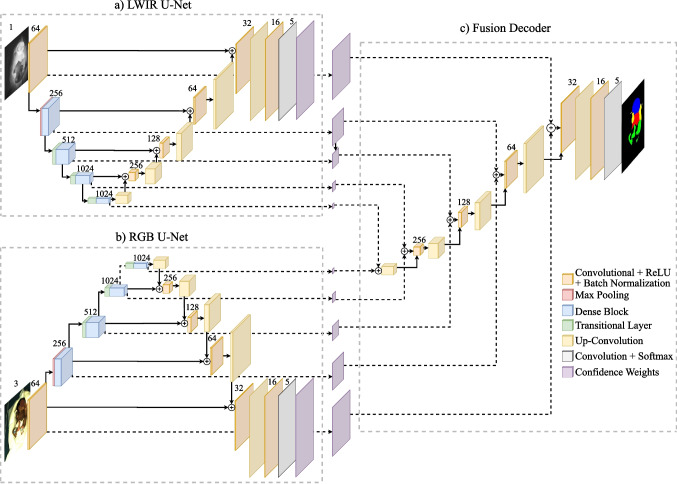
The confidence fusion U-Net architecture with **a**) an LWIR U-Net; **b**) an RGB U-Net; **c**) the decoder fusing the weighted features of both encoders

Two models used unimodal image data: one processing RGB images only, the other processing LWIR images only. These unimodal models serve as an important baseline to assess the effectiveness of the fusion strategies.

While the unimodal models rely on basic U-Nets with an encoder-decoder structure, an additional model integrates multimodal image information through a feature fusion technique. This fusion model applies a confidence weighting approach. Adopted from [22], this approach is shown in Fig. 3.

It includes two separate U-net models for RGB and LWIR images and an additional shared decoder. The confidence weighting concept considers that different modalities may be more appropriate for segmentation depending on factors such as lighting conditions, incubator temperature and the infant’s skin temperature. For example, in poor lighting conditions, LWIR images are more suitable than RGB images. To account for these conditions, a confidence matrix is used to adapt to each situation. After the softmax activation layer, the output of the unimodal networks provides the probabilities for each class at each pixel location. This information is used to derive the confidence matrix, where an entry $C_{ij}$ represents the highest probability value at pixel (*i*, *j*). In particular, it reflects the network’s confidence in its prediction for that particular pixel. The confidence matrix is then scaled to match the spatial dimensions of the encoder’s feature maps. The feature maps of the unimodal networks are weighted and merged with the confidence matrix by element-wise multiplication. Both element-wise addition and concatenation were analyzed to combine the weighted unimodal features. The loss function and training procedure used to optimize this architecture are detailed in the following section.

#### Training procedure

As previous works [10, 12] demonstrated the success of data augmentation and transfer learning for adult datasets, we also applied these methods to mitigate the effects of the relatively limited dataset size resulting from the manual annotation process. After extensive testing, five specific image augmentations were selected to prevent overfitting of the neural networks on the relatively small infant dataset. These augmentations include rotating images up to 30$^{\circ }$, scaling images between 0.8 and 1.2, shifting images horizontally and vertically, flipping images horizontally, and applying coarse dropout to mask rectangular regions. Transfer learning was investigated using the CIHP dataset [13]. This dataset contains 38280 manually labeled images, where we merged the classes into head, torso, arm, leg, and background.

In addition, cross-validation was performed to evaluate the body part segmentation. The dataset was divided into four equal-sized folds for cross-validation and an additional test set for a final evaluation afterwards. Each fold contained between 197 and 199 images, consisting of two to four infants, while the final test set consisted of 167 images. All images from an individual infant were always assigned to the same fold. The dataset split is detailed in Table 3.

**Table 3:** Division of the dataset for cross-validation

	IDs	Number of images
Fold 1	PAT05, PAT11	198
Fold 2	PAT01, PAT02, PAT03, PAT08	198
Fold 3	PA0T4, PAT06, PAT07	199
Fold 4	PAT09, PAT10	197
Test-Set	PAT12, PAT13, PAT14	167

For training, the categorical cross-entropy was used as the loss function. For confidence fusion, the losses of the unimodal parts and the decoder are determined and then added together. In addition, the Adam optimizer was selected with the coefficients $\beta _1$=0.9 and $\beta _2$=0.999. In total, the networks were trained for 30 epochs with a learning rate of 0.0001.

#### Evaluation metrics

To accurately extract the temperature, the segmentation must be as accurate as possible, containing only correct pixels (true positives) of the corresponding class, while minimizing incorrectly classified pixels (false positives), such as background pixels. The higher the number of correct pixels extracted by the segmentation, the more robust the subsequent temperature extraction. Therefore, IoU and precision were chosen as the metrics to evaluate the segmentation. IoU indicates the overlap between the predicted region and the ground truth region, divided by their union, while precision measures the correctness of the prediction.

### Real-time temperature correction

Achieving accurate absolute temperature measurements with IRT requires careful consideration of a number of influences. This is particularly critical when measuring the temperature of a premature infant in an incubator, as the closed environment adds complexity to the measurement. An IRT camera outside the incubator captures thermal radiation through an IR-transparent window. However, this window, along with other factors inside the incubator, significantly affects the radiation received by the camera. Previous research [4, 23] has investigated compensation methods for these influences. However, these authors did not carry out a robust investigation with a sufficient amount of measurement data. Similar to previous research, thermal reference sources are typically used to calibrate infrared cameras in real-time to ensure compensation of unwanted influences.

**Fig. 4:**
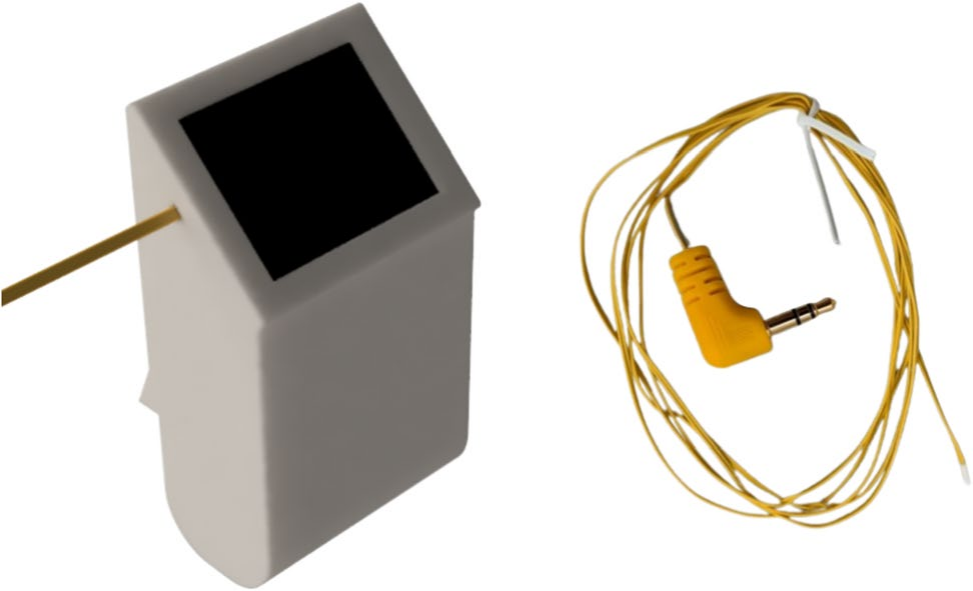
Custom passive thermal reference (left) with clinically used temperature sensors (right)

For this work, a custom-built passive thermal reference source was placed inside the incubator within the camera’s field of view. The thermal reference setup is depicted in Fig. 4.

We chose an NTC thermistor (Weyer GmbH), commonly used for clinical measurement of infant skin temperature. This sensor offers high accuracy (± 0.1 $^{\circ }$C) and its readings can be accessed from the incubator’s data output, as the incubator’s integrated system handles the necessary data acquisition. It was integrated into a silicone housing made by casting SILIXON10 (silikonfabrik.de) into a 3D printed mould. The top of the housing (surface area 3.5 $\times$ 8 cm) was covered with Acktar Metal Velvet Black Foil (Acktar Ltd., Israel), which provides a high, diffuse emissivity ($\epsilon >0.98$) in the long-wave infrared spectrum, closely matching the emissivity of human skin. The NTC thermistor is positioned in direct contact with the underside of the foil to ensure that the measured temperature accurately represents the radiating surface temperature, minimizing thermal gradients.

#### Calibration algorithm

During the recordings, it was noticed that the infrared window reflects some of the IRT camera’s thermal radiation, which interferes with the measurements. After a warm-up period, this effect becomes a continuous additive disturbance that can be compensated for by a non-uniformity correction (NUC). To correct for non-uniformity, we utilized a custom calibration target consisting of a 3D-printed PLA carrier structure (dimensions: 20 x 20 cm) featuring a flat surface. This surface was covered with the same high-emissivity Acktar Metal Velvet foil used for the thermal reference. The target was placed inside the incubator at a stable air temperature of 32 $^{\circ }$C and allowed to thermalize for 30 minutes. The high thermal conductivity of the foil’s aluminum substrate, combined with the stable isothermal environment, ensured a uniform radiation pattern across the surface. The average of 15 images was calculated to correct for possible noise, allowing the correction matrix to be determined as:

1 $$\begin{aligned} T_{\text {NUC, Mat.}}(x,y)= \frac{1}{15}\sum _{n=1}^{15}(T_{\text {cam,surf}}(x,y)(t_n)-T_{\text {ref,surf}}(t_n)) \end{aligned}$$

Here, $T_{\text {NUC, Mat.}}$ is the resulting pixel-wise correction matrix. For each image in the sequence, $T_{\text {cam,surf}}$ represents the temperature measured at an individual pixel (x,y). $T_{\text {ref,surf}}$ is the known, uniform temperature of the calibration surface (a single scalar value). The correction term for each pixel is found by subtracting this scalar reference temperature from the pixel’s measured value. The corrected infrared image is then given by:

2 $$\begin{aligned} T_{\text {cam, corr.}}(x,y)(t) = T_{\text {cam}}(x,y)(t) - T_{\text {NUC, Mat.}}(x,y) \end{aligned}$$

Furthermore, other influences also interfere with the temperature measurement. These have been compensated for using the following correction equation:

3 $$\begin{aligned} T_{\text {Temp,Corr}}= a_{\text {0}} + a_{\text {1}}( T_{\text {Temp,IRT}}-a_{\text {2}} (T_{\text {Ref,IRT}}-T_{\text {Ref,Sens}})) + a_{\text {3}} \cdot T_{\text {Ref,Sens}}, \end{aligned}$$

which is based on the IRT-measured temperature $T_{\text {Temp,IRT}}$, the IRT-measured thermal reference temperature $T_{\text {Ref,IRT}}$ and the sensor-based thermal reference temperature $T_{\text {Ref,Sens}}$ with $a_{\text {0}}$, $a_{\text {1}}$, $a_{\text {2}}$, $a_{\text {3}}$ being fitted coefficients. The correction algorithm incorporates the combined effects of drift, environmental factors and incubator air temperature.

#### Accuracy evaluation

**Fig. 5:**
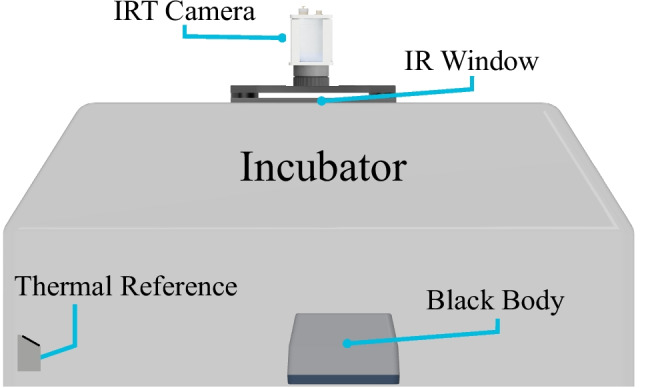
Measurement Setup including blackbody and thermal reference

The measurement setup for accuracy calibration can be seen in Fig. 5. An active black body (‘Nightingale Body Temperature Reference’, Santa Barbara Infrared Inc., Santa Barbara, USA) was placed inside the incubator as the measuring object. The emissivity of this measuring object is 95 % and is therefore similar to the emissivity of human skin [24]. The measuring object can achieve temperatures with an accuracy of ±0.15 $^{\circ }$C. In addition, the incubator contains the same thermal reference as in the clinical study. A total of 26 series of measurements were taken, each lasting seven hours, with the position of the black body altered over the whole incubator mattress. The air temperature in the incubator was set between 31 $^{\circ }$C and 37 $^{\circ }$C for each recording. For each set air temperature, the black body temperature was varied between 35 $^{\circ }$C and 40 $^{\circ }$C to cover all clinically relevant combinations of incubator air temperature and infant skin temperature.

The 26 measurements were divided into five equally sized folds, with a complete series of measurements assigned to each fold. The calibration algorithm’s coefficients were determined using the Levenberg-Marquardt algorithm with a least-squares cost function to optimize the model described in Eq. 3. The high data-to-parameter ratio (26 long-term recordings for 4 coefficients) ensures a robust fit without the need for complex regularization. Furthermore, external radiative sources (e.g., the infant) are accounted for by the high thermal conductivity of the foil substrate, which prevents significant thermal gradients between the exposed surface and the rear-mounted sensor.

The exact values of the fitted coefficients are highly dependent on the specific hardware configuration, including the camera model, incubator geometry, and the properties of the infrared window. As these values are not generalizable to other setups, they have been omitted.

For validation, the mean absolute error (MAE), maximum error (MAX), and standard deviation (STD) of the IRT temperature measurements were determined, both before (raw) and after (corrected) applying the calibration algorithm. The evaluation results are presented in Table 4.

**Table 4:** Qualitative results of temperature correction for one measurement

Metric	Raw	Corrected
Mean absolute error [$^{\circ }$C]	0.91	0.17
Max. Error [$^{\circ }$C]	4.85	0.83
Standard deviation [$^{\circ }$C]	0.79	0.12

We note that the presented calibration method significantly improves the accuracy of the temperature measurement, reducing the measurement error by 81.3 %. In addition, the maximum error was reduced from 4.85 $^{\circ }$C to 0.83 $^{\circ }$C, yielding a reduction of 83 %.

### Spatial temperature extraction

**Fig. 6:**
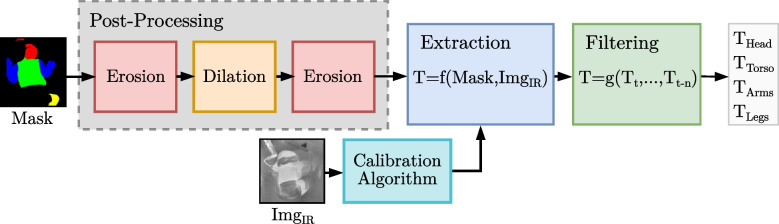
Temperature extraction process including post-processing, temperature extraction and filtering

With the segmentation masks generated by the neural network, the temperature can be extracted. First, the masks were transformed to align with the original LWIR images, followed by several post-processing steps to improve the precision. Next, the calibration algorithm from Section 2.4 was applied to the LWIR image. Finally, we analyzed specific methods to extract and filter the temperature: percentile-based extraction to minimize outliers and a Kalman filter to ensure temporal stability. Figure 6 illustrates the schematic process of the temperature extraction algorithm.

**Fig. 7:**
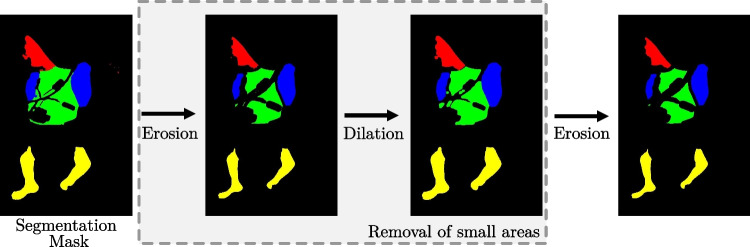
Example of post-processing: Removal of small regions with subsequent separate erosion

To increase the precision of the segmentation masks, a uniform and automated post-processing pipeline was applied to all images, as illustrated in Fig. 7. This process consisted of two main stages. First, to automatically remove small, erroneous regions, a morphological opening operation (an erosion followed by a dilation) was performed. For this, a square kernel of size 10x10 pixels was used. Subsequently, to ensure no background pixels were included at the boundaries due to minor misalignments, a final, separate erosion was applied using a smaller kernel of size 8x8 pixels. These kernel sizes were determined empirically and were kept constant for all images from every infant to ensure consistent processing. The post-processing pipeline is illustrated in Fig. 7.

After post-processing, the temperature was extracted based on the pixels of the LWIR image assigned to a class by the segmentation mask. Different mathematical methods for the final temperature calculation were investigated and compared with the reference sensor attached to the torso. The analyzed approaches included averaging and percentile-based thresholding.

In the case of percentile thresholding, the upper and lower percentiles of the temperature values per class were omitted for further extraction. For example, in the lower 10/upper 10 percentile approach, the 10 % lowest and 10 % highest values were excluded from the temperature extraction. More demanding percentiles such as lower 50/upper 10 were also examined. The final temperature was determined by averaging the remaining values.

To achieve stable temperature measurements, the extracted temperatures were further refined using a Kalman filter. As thermoregulation is a gradual process, no fast changes in temperature readings were expected. It was also assumed that the temperature in each of the four regions (head, torso, arms, legs) was not influenced by any additional known inputs. All temperature variations were modeled as process noise with zero mean and assumed variance. This also applies to the observed space. For the process noise *Q* it was assumed that a change in skin temperature of 0.1 $^{\circ }$C per min is possible. This corresponds to a change of 1 $^{\circ }$C per  10 min and led to a process noise of $Q_{\text {Kalman}} = \frac{0.1}{60}^2$. The standard deviation of the LWIR camera after calibration was used for the measurement noise. This resulted in a measurement noise of $R_{\text {Kalman}} = 0.12^2$ for the Kalman filter.

#### Evaluation metrics

Different metrics were defined to evaluate the temperature extraction process quantitatively. The skin temperature sensor attached to the infant’s torso during recording served as the reference for these metrics, although it only provides information about the torso.

The mean absolute, median and maximum errors were used to measure the accuracy relative to the skin temperature sensor. In addition, the moving standard deviation (movSD) was chosen to assess the robustness of the extracted temperature itself. As temperature measurement is a non-stationary process, with body temperature fluctuating over time due to metabolic and environmental factors, a standard deviation would not capture these gradual changes and could provide misleading information about the stability of the measurements.

Therefore, the averaged movSD with a window size of 200 seconds was used. This metric identifies short-term fluctuations in temperature readings and provides a clearer picture of the consistency and reliability of the temperature extraction process. For example, relatively stable temperature readings with small variations within each window indicate a robust extraction method, whereas significant variability indicates noise or inaccuracy.

Because the preterm infants were monitored for periods of more than five hours, during which care activities such as diaper changes occurred, accurate temperature readings were not possible when the infants were partially or fully covered. To ensure the reliability of the analysis, metrics were calculated only for periods without care activities.

## Results

### Segmentation results

To identify the most effective segmentation approach, four-fold cross-validation was performed on the training dataset, evaluating different model architectures and training configurations. This initial evaluation led to the selection of optimal models for both unimodal and fusion approaches, which were then tested on a separate test set to determine their generalization performance.

#### Cross-validation

**Fig. 8:**
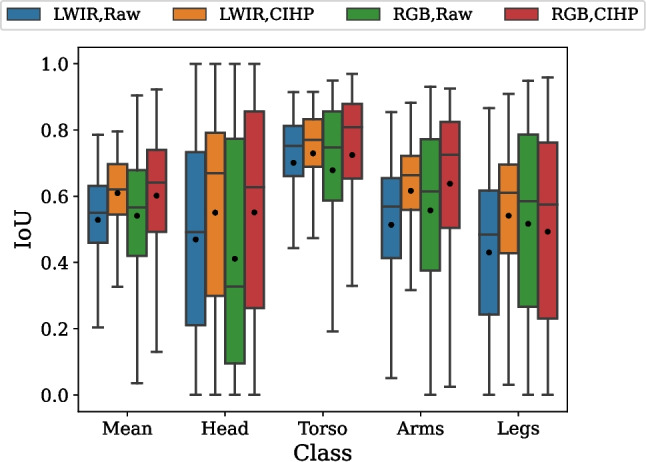
IoU for all classes during cross-validation of the unimodal LWIR and RGB models, comparing results without pre-training (Raw) and with pre-training (CIHP)

The results for the unimodal RGB and LWIR networks, both without transfer learning (Raw) and with transfer learning using the CIHP dataset, are presented in Fig. 8.

As can be seen in the box plot, models using pre-training consistently achieved higher IoU values in both modalities. The pre-trained RGB model achieved an mIoU of 0.6, outperforming the RGB model without transfer learning (mIoU of 0.54). A similar trend is observed for the LWIR modality, where the pre-trained model achieved an mIoU of 0.61, outperforming the model without transfer learning (mIoU of 0.53). In addition to IoU, we also evaluated segmentation performance using precision, as demonstrated in Table 5.

**Table 5:** Precision scores for unimodal models

Modality	Pre-training	Mean	Head	Torso	Arms	Legs
LWIR	-	0.72	0.65	0.81	0.70	0.69
LWIR	CIHP	0.75	0.66	0.83	0.76	0.76
RGB	-	0.77	0.65	0.87	0.75	0.79
RGB	CIHP	0.80	0.76	0.86	0.80	0.80

The pre-trained RGB model achieved an average precision of 0.80, while the raw RGB model achieved 0.77. This improvement due to pre-training was particularly evident for the head region, where precision increased from 0.65 to 0.76. The LWIR modality gave a similar pattern, with the pre-trained model achieving an average precision of 0.75 compared to 0.72 for no pre-training.

Across all configurations, the torso consistently achieved the highest IoU and precision, followed by the arms. The legs performed relatively well, except in the LWIR training without transfer learning. The head remained the most difficult body part to segment, consistently exhibiting the lowest IoU and precision, even with the benefit of pre-training.

**Fig. 9:**
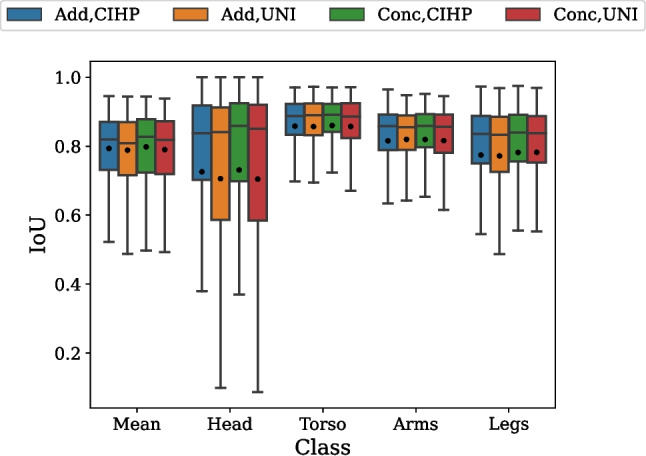
IoU for all classes during cross-validation of the fusion models with element-wise addition (Add) or concatenation (Conc), comparing results CIHP pre-training and using weights from the best performing unimodal network (UNI)

Building on the unimodal results, we then explored the benefits of incorporating pre-training into our fusion models. Two fusion strategies were employed: element-wise addition (Add) and concatenation (Conc) of unimodal features. For each, the models were pre-trained using either the CIHP dataset or the weights from the best performing unimodal network (UNI). Figure 9 displays the results of the cross-validation. Both fusion methods achieved comparable overall performance. The model using feature concatenation, pre-trained on the CIHP dataset, achieved the highest mean IoU of 0.80, closely followed by the model using element-wise addition, also pre-trained on CIHP, which achieved a mean IoU of 0.79. The configurations using weights taken from the best unimodal network performed slightly lower, with both models achieving a mean IoU of 0.78. We also evaluated segmentation performance for the fusion models using precision, as presented in Table 6.

**Table 6:** Precision scores for fusion models

Fusion Method	Pre-training	Mean	Head	Torso	Arms	Legs
Addition	CIHP	0.87	0.79	0.90	0.89	0.90
Addition	UNI	0.87	0.77	0.91	0.90	0.91
Concatenation	CIHP	0.88	0.80	0.91	0.90	0.90
Concatenation	UNI	0.86	0.76	0.90	0.90	0.89

Overall, the fusion models achieved high precision scores in all configurations. The models using CIHP pre-training generally performed slightly better than those initialized with UNI weights. For both element-wise addition and concatenation, CIHP pre-training resulted in mean precision scores of 0.87 and 0.88, respectively, while UNI pre-training resulted in slightly lower scores of 0.87 and 0.86. However, these differences are relatively small, suggesting that both pre-training strategies produce comparable performance.

Across all fusion models and pre-training configurations, the torso consistently achieved the highest performance for both IoU and precision. The arms and legs also performed consistently well, with IoU and precision scores generally above 0.77 and 0.89 respectively. In contrast, the head remained the most difficult body part to segment. While pre-training on the CIHP dataset resulted in a slight improvement in head segmentation, with IoU increasing to 0.73 (Conc, CIHP) and precision to 0.80, the head still performed below other body parts.

#### Validation on test set

**Fig. 10:**
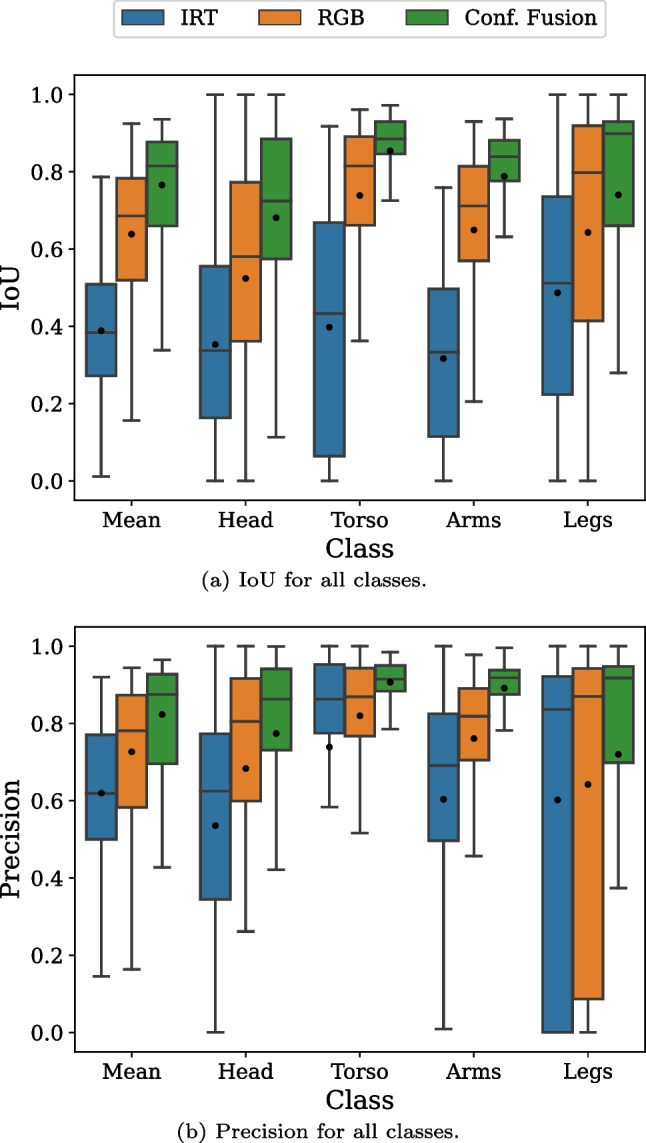
Results on the test set for the optimized models

After cross-validation identified the best model configurations, they were evaluated on a withheld test set. The cross-validation folds served as training data, and the results on the test set are shown in Fig. 10.

In the test set, the LWIR model (CIHP pre-trained, CLAHE-processed images) demonstrated an mIoU of 0.39 and a precision of 0.62. The most effective RGB-only model (also CIHP pre-trained) demonstrated an mIoU of 0.64 and a precision of 0.73. A straightforward combination of these unimodal models resulted in an mIoU of 0.76 and a precision of 0.81. The confidence fusion model (CIHP pre-trained) demonstrated the best performance, achieving an mIoU of 0.766 and a precision of 0.82, resulting in the best overall model.

**Fig. 11:**
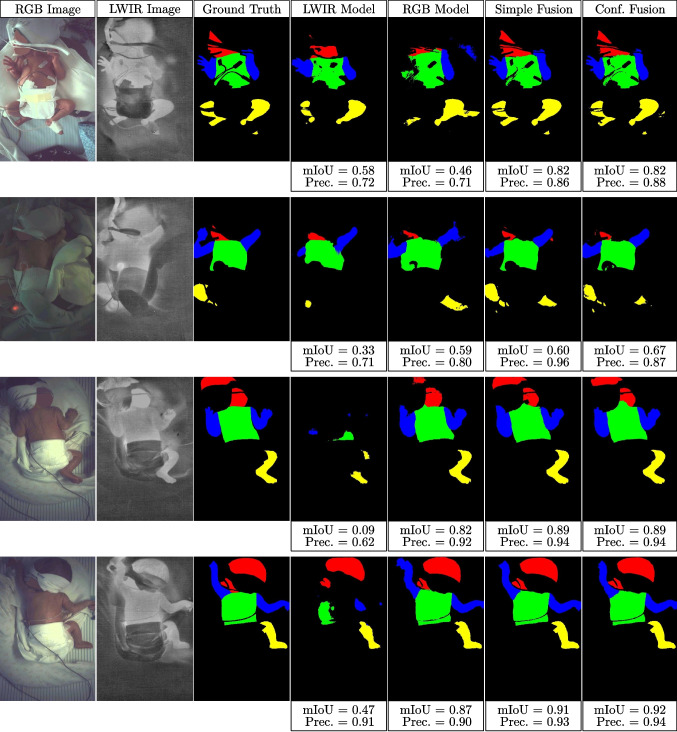
Qualitative results of four sample images from the test set

The head was the most challenging part of the body to segment, while the torso demonstrated the highest accuracy. Notably, the confidence fusion model exhibited lower accuracy in segmenting the legs than the simple fusion model. Surprisingly, the LWIR model’s performance on the test set was significantly inferior to its cross-validation results. To help understand the results, qualitative examples of segmentation are provided in Fig. 11.

The results for the LWIR network, as illustrated in rows three and four of Fig. 11, clearly demonstrate its limitations. Qualitative analysis of the model’s predictions reveals poor performance in segmenting the arms and torso, consistent with the quantitative results in Fig. 10. The remaining examples in Fig. 11 provide visual evidence confirming the superior performance of the fusion models.

### Spatial temperature extraction

This section presents the results of the temperature extraction. Since the confidence fusion approach provided the best results, it was used to segment the body parts for subsequent temperature extraction. As a first step, the post-processing of the generated segmentation masks was evaluated. Then the results of the temperature measurements were examined.

**Fig. 12:**
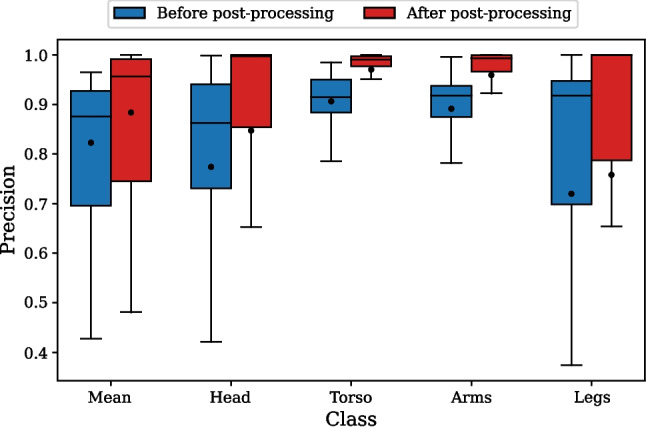
Precision comparison of all classes before and after post-processing

Figure 12 depicts the precision for all classes before and after post-processing.

Overall, the mean precision of the test set increased from 0.82 to 0.88. Post-processing significantly improved the precision for torso and arms segmentation, with the torso precision increasing from 0.91 to 0.97 and the legs having the smallest improvement from 0.72 to 0.76.

With the improved masks, the temperature was extracted with the pipeline depicted in Fig. 6. The validation process was carried out in two stages. First, the extracted torso temperature was validated against the recorded reference sensor to identify the best extraction method (averaging, percentiles). Then the temperature extraction was compared across all body regions.

As the reference skin sensor was attached to the liver region of the torso, temperatures from the head, arms and legs were not used to determine the best extraction method. Both quantitative analysis and qualitative evaluation were used to assess the accuracy and agreement of the methods.

**Fig. 13:**
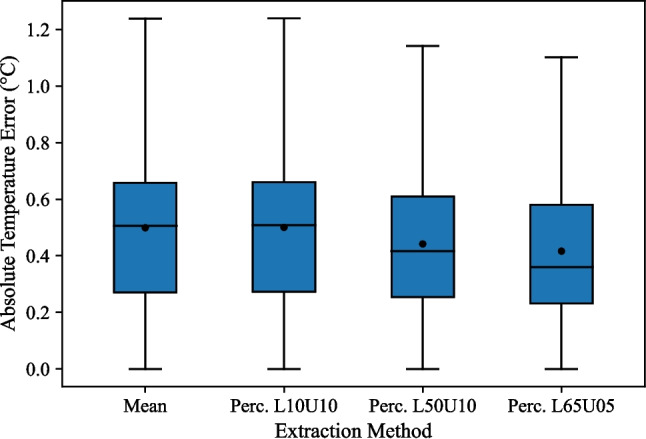
Comparison of temperature error for different extraction methods averaged over all recorded patients

Figure 13 shows a quantitative analysis using box plots, averaged over all recorded infants. Four methods were compared: calculating the mean temperature, excluding different percentiles such as the lower and upper 10th percentile (L10U10), the lower 50th and upper 10th percentile (L50U10), and the lower 65th and upper 5th percentile (L65U05). Other methods were also investigated but resulted in lower accuracies and were therefore not investigated further.

The Mean and Percentile L10U10 methods demonstrate similar error distributions, with mean errors around 0.5 $^{\circ }$C and median errors of 0.51 $^{\circ }$C. In contrast, the Percentile L50U10 and L65U05 methods, which use higher percentile ranges, provide slightly improved accuracy. These methods achieve lower mean errors of 0.44 $^{\circ }$C and 0.41 $^{\circ }$C respectively, with median errors of 0.42 $^{\circ }$C and 0.36 $^{\circ }$C respectively. The box plots visualize these results, illustrating a slight reduction in the median error and a narrower interquartile range for the higher percentile methods.

**Fig. 14:**
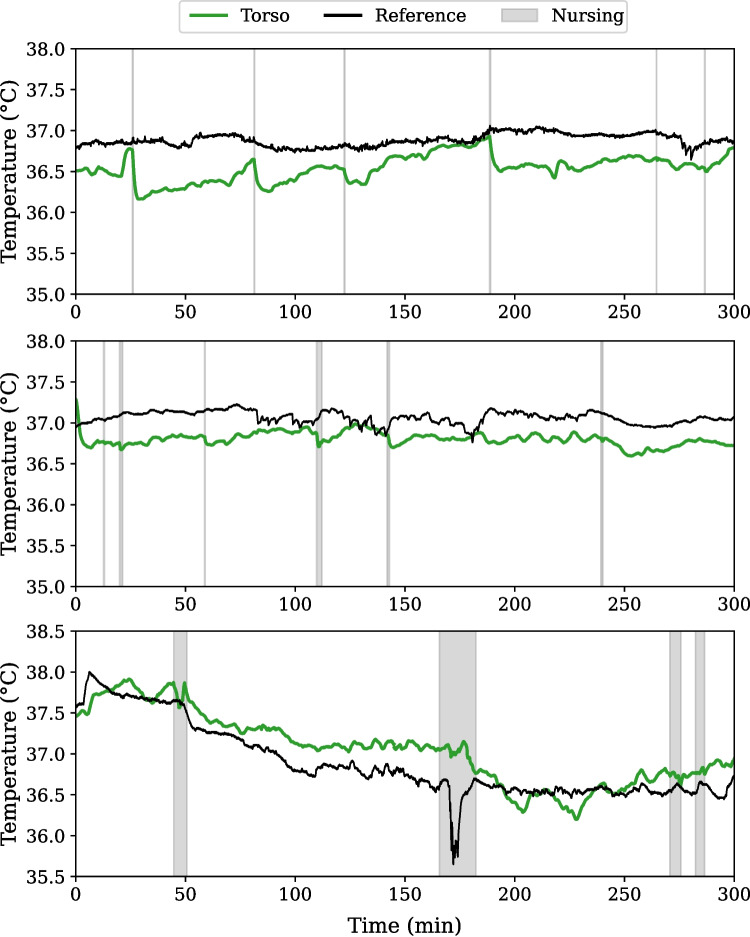
Extracted temperatures of the torso for three recorded infants compared to adhesive skin sensor as reference

In addition, a qualitative evaluation was performed to verify the performance of the L65U05 method, which gave the best quantitative results. The extracted torso temperature, determined by excluding the lower 65th and upper 5th percentiles, is compared to the reference temperature in Fig. 14. For better visibility, only three recordings are presented, with the gray shaded areas representing the time intervals during which nursing care was provided.

Figure 14 presents a qualitative comparison, demonstrating that the extracted torso temperatures successfully capture the overall thermal dynamics of the reference temperatures. For instance, a general cooling trend is evident in the bottom plot for both measurement types.

However, notable differences emerge upon closer inspection. The IRT-based measurement appears more volatile than the reference in the top plot, yet more stable in the middle and bottom plots. Moreover, the absolute torso temperatures frequently deviate from the reference values by as much as 0.5$^{\circ }$C.

Both measurement techniques also register short-term fluctuations, caused primarily by nursing activities. This is particularly clear in the lower plot, where the reference sensor shows a significant temperature drop after approximately 170 minutes. A unique artifact is also present in the middle plot, which displays an unexpected, transient drop in the beginning of measured torso temperature that is not present in the other plots.

While the torso temperature provides a valuable overall assessment, the accuracy of IRT-based temperature measurements can vary significantly between different body regions due to factors such as skin visibility, motion artifacts and proximity to heat sources. Therefore, the next step was to compare temperatures obtained from different parts of the body (arms, legs, head) to determine which regions provided the most reliable and robust estimates of central and peripheral temperatures.

**Fig. 15:**
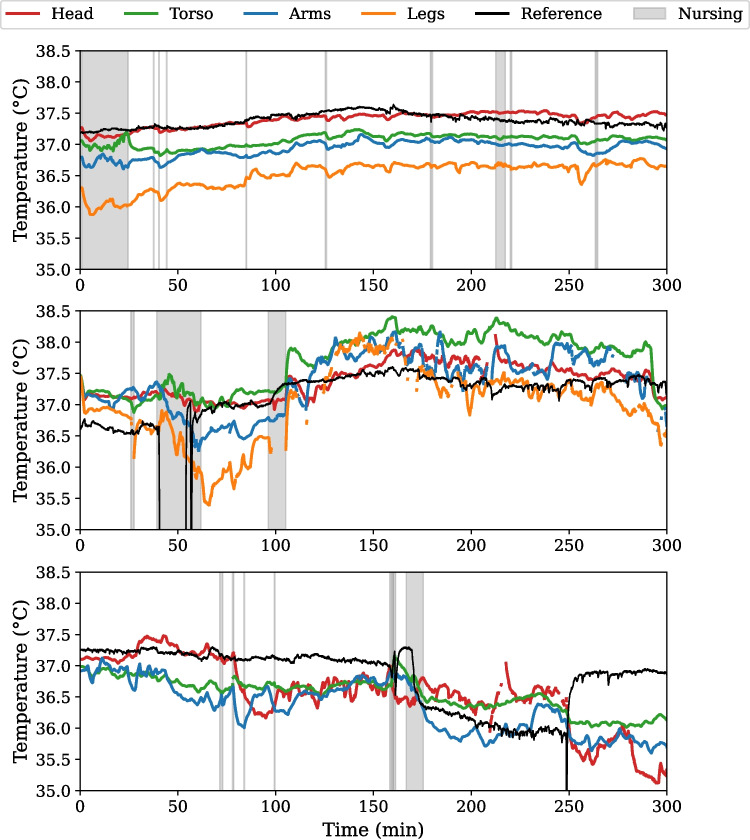
Extracted temperatures of all body parts for three recorded infants with adhesive skin sensor as reference

Both qualitative evaluation and quantitative analysis were used to assess the accuracy and robustness of temperature extraction for each region. For illustration, Fig. 15 provides the extracted temperatures from all body parts, along with the measured temperature of the adhesive skin sensor for reference. Here, three different recordings are presented, with the gray shaded areas representing the time intervals during which nursing care was provided.

The extracted temperatures for all body regions were within typical skin temperature ranges. However, there was no extracted legs temperature for the bottom plot since the infant’s legs were covered throughout the recording. As expected, the torso and head had consistently higher temperatures than the arms and legs, in line with their closer proximity to core body temperature. Specifically, the difference in skin temperature between peripheral and central body regions in preterm infants is generally between 0.5 $^{\circ }$C and 1 $^{\circ }$C [25].

While temperature variations were observed in all regions, the head and legs had greater variations compared to the torso and arms. As shown in Fig. 15, the extracted temperatures generally follow the trend of the reference sensor, though with distinct regional offsets.

To provide a more comprehensive assessment of the reliability of the extraction method, both the movSD and the percentage of time that each body region was undetectable were examined. Table 7 presents the movSD of the extracted temperatures, calculated using a 200-second sliding window and averaged across all recorded infants, for each body region (head, torso, arms, legs). In addition, the table includes the percentage of time that each region was not detected during the recordings, providing a measure of the availability of each region for consistent monitoring.

**Table 7:** MovSD and non-detection rate of extracted temperature per class, averaged over all recordings

Metric	Head	Torso	Arms	Legs
movSD ($^{\circ }$C)	0.032	0.021	0.032	0.032
Undetectable (%)	4.2 %	0.1 %	1.6 %	10.7 %

The torso region demonstrated the lowest movSD (0.021 $^{\circ }$C), indicating less temperature variation compared to all other body parts, which had similar movSD values. Importantly, the torso was also the most consistently detected region, with measurements available in 99.9 % of the recordings. In contrast, the legs were the least consistently detected, with new temperature measurements unavailable in 10.7 % of recordings, necessitating the use of the last recorded value when not detected. The head and arms showed intermediate detectability at 95.8 % and 98.4 % respectively.

## Discussion

### Segmentation

Our final segmentation model achieved a mean Intersection over Union (mIoU) of 0.77. This result compares favorably with the state-of-the-art, showing an improvement over the 0.70 mIoU reported by Asano et al. [11]. While the performance is lower than the 0.87 mIoU achieved by Voss et al. [12], it is important to note that our data was collected without the enhanced, controlled lighting used in the previous study, representing a more challenging and realistic clinical scenario.

Our cross-validation experiments revealed that pre-training significantly improved segmentation accuracy for both unimodal networks, confirming the findings of Voss et al. [12] and Antink et al. [10]. Analysis of individual class performance revealed the torso to be the most accurately segmented body part, with consistently superior results across all RGB and LWIR model configurations. This superior performance can be attributed to the distribution of pixels across classes in the training dataset, as detailed in Table 8.

**Table 8:** Percentage distributions of the classes of the dataset

	Background	Head	Torso	Arms	Legs
Percentage	83.91 %	4.00 %	5.77 %	3.82 %	2.51 %

In contrast to the work of Voss et al. [12], the dataset of this work has a higher proportion of pixels labeled as torso (5.77 %) compared to legs (2.51 %). This difference in class distribution may account for the superior torso segmentation results observed in this study, in contrast to the significantly poorer torso segmentation reported in [12]. This class imbalance can lead to biased model performance and reduced generalisation, making accurate scoring difficult. To mitigate this, strategies such as data augmentation, class rebalancing, and synthetic data generation can be employed to ensure more fair representation across all classes.

**Fig. 16:**
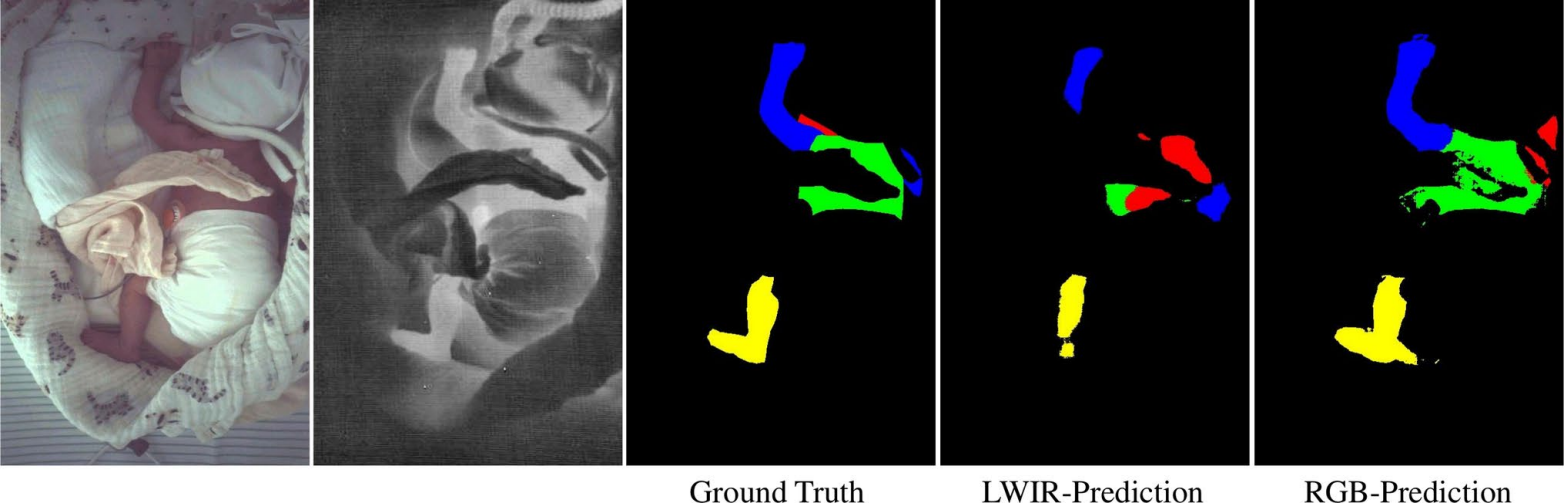
Example of the head segmentation by the unimodal networks

In addition, both unimodal networks had the lowest IoU and precision scores for the head class, indicating a high rate of misclassification. This may be due to the presence of head coverings or respiratory masks on the infants, which are difficult to distinguish from the infant’s skin under poor lighting or similar thermal conditions. Figure 16 provides a visual example of this challenge.

This image pair shows a infant hat that almost completely covers the head. The RGB image suffers from poor illumination in this region, while the hat appears bright in the LWIR image. The failure of the unimodal networks to correctly segment the head in this case supports the hypothesis that head coverings pose a significant challenge to accurate head segmentation.

While the cross-validation results of the LWIR model were comparable to those reported in the literature [11, 12], its performance on the final test set was significantly lower. This discrepancy could be attributed to the uneven heat distribution of some body parts within the LWIR images. As illustrated in Fig. 11, the torso and arms have dark and light regions, with the darker areas indistinguishable from the background. As a result, the model accurately identified only the lighter regions. Furthermore, the performance of the RGB model was also significantly worse than reported in the literature [10, 12], likely due to the lack of additional lighting during data acquisition. In contrast, previous studies reporting superior results used images with enhanced contrast and brightness provided by external light sources.

The results clearly indicate that fusing RGB and LWIR image data within a neural network significantly improves and stabilizes segmentation performance compared to using either modality alone. Even with the suboptimal performance of the LWIR model, the multimodal fusion approach significantly outperforms the RGB model. In particular, the confidence fusion method proved to be more effective than simple feature concatenation in incorporating multimodal information for robust body part segmentation. The superiority of confidence-based fusion over simple concatenation suggests that maintaining separate feature extraction paths allows the model to better filter modality-specific noise. By weighting predictions based on pixel-wise certainty, the system can dynamically reject unreliable data (e.g. thermal artifacts) in favor of the more stable modality, rather than merging potentially conflicting features early in the network.

While the presented results are promising, there are several areas for future improvement. The current manual image registration process, which involves selecting individual RGB-LWIR image pairs, is a significant limitation. This manual approach is inefficient and unsuitable for the ultimate goal of automated temperature measurement. Therefore, an automated registration method is desirable for future development. In addition, the current hardware setup is not ideal for integration into a clinical setting. A more compact and less intrusive design, seamlessly integrated into the incubator, would significantly improve usability and reduce interference during medical procedures or neonatal care. The relatively small dataset size (959 image pairs) also is a limitation. While we mitigated this through transfer learning (CIHP) and standard geometric data augmentation, the class imbalance (e.g., legs 2.51 %) remains a challenge. Future work could address this by expanding the dataset through synthetic data generation techniques [26] to ensure more robust generalization across different demographics and hardware setups. This dataset size limits the exploration of more complex and potentially more accurate deep learning architectures, such as those based on transformers. Expanding the dataset, perhaps by including synthetically generated data, would allow for a more thorough investigation of such architectures and could lead to significant gains in segmentation accuracy and robustness.

### Temperature extraction

The accuracy of our spatial temperature measurements is built upon two key stages: first, the correction of the raw thermal data, and second, the extraction of temperature from the segmented body parts. A core component of our system is the temperature correction algorithm, which achieved a mean absolute error (MAE) of 0.17$^{\circ }$C in blackbody validation tests. This represents a significant improvement in foundational accuracy over previously reported methods, such as the sub-0.3$^{\circ }$C MAE described by Hamada et al. [4]. This is particularly significant as our system operates with the camera outside the incubator. In contrast, Hamada et al.’s approach involved placing cameras inside the incubator, which bypasses the significant challenge of correcting for errors introduced by an infrared window.

This high-accuracy corrected image, obtained under more demanding conditions, serves as the input for the subsequent temperature extraction process. The final clinical accuracy is therefore dependent not only on this initial correction but also on the specific method used to derive a representative temperature from the pixels within a segmented body part. The results of the quantitative analysis in Fig. 13 and the qualitative evaluation in Fig. 14 strongly suggest that the use of higher percentiles in temperature extraction methods, such as Percentile L50U10 and L65U05, significantly improves measurement accuracy compared to simple averaging-based methods. This improvement is evident from the lower mean and median errors and reduced variability observed in the box plots. The use of higher percentiles effectively reduced the effect of outlier pixels, resulting in a more reliable temperature estimate with an overall mean error of approximately $0.41^{\circ }C$. While wider than contact sensors, this error margin is sufficient to reliably detect key clinical trends, such as the onset of cold stress ($>0.5^{\circ }C$) or changes in the central-peripheral temperature gradient ($0.5-1.2^{\circ }C$).

However, there is still room for improvement. Although high precision was aimed for during segmentation, subsequent processing inadvertently reduced the size of the regions of interest (ROIs), potentially compromising temperature extraction accuracy. These smaller ROIs may be more susceptible to noise and less representative of the overall body part temperature.

In addition, uncompensated thermal radiation from the interior of the incubator and unknown reflections from the IR window introduce potential systematic errors. A potential solution could be to integrate long-wave infrared (LWIR) cameras directly inside the incubator, for example in the upper corners, to eliminate IR window interference and improve accuracy.

Furthermore, mapping the spatially derived torso temperature to the single-point reference measurement in the liver region is challenging, especially in prone-lying infants. This is illustrated by an observation in the results (see Fig. 15), in which the spatially averaged head temperature is sometimes more aligned with the reference sensor than the spatially averaged torso temperature. This counterintuitive finding can be explained by several factors. First, the reference is not an accurate representation of the average torso temperature; rather, it is a single-point measurement from the metabolically active liver region, which acts as a thermal hot spot. In contrast, our IRT-based method averages the temperature over the entire visible torso surface, which is expected to differ from the hot spot reference. Second, the infant’s position is crucial; in a prone infant, the camera measures the back, while the reference sensor on the abdomen is insulated, naturally creating a discrepancy. Third, the head itself is a region of high, stable temperature due to cerebral blood flow. This means its average surface temperature maytrack the core-adjacent liver hot spot more closely than the average of the cooler, larger torso surface.

These findings raise the question of whether a spatially resolved reference or multiple sensor locations would provide a more suitable ground truth for assessing the accuracy of thermal imaging. Future work should address these limitations by exploring larger, more robust regions of interest (ROIs), implementing methods to compensate for incubator radiation, and investigating alternative ground truth measurement strategies.

The results in Fig. 15 show significant temperature fluctuations, particularly in the middle and bottom plots. By cross-referencing these timestamps with the synchronized RGB video recordings, we visually confirmed that these fluctuations coincide directly with neonatal care interventions (e.g. diaper changes, examinations). This visual verification confirms that the observed deviations are physical thermal responses to handling—such as the cooling of exposed skin or the temporary occlusion of the reference sensor, rather than artifacts caused by radiative coupling changes in the reference system.”

The body part comparison revealed that the torso consistently had the lowest temperature variation, as indicated by the moving standard deviation and the highest detection rate. This, combined with its relatively larger pixel fraction in the dataset, established the torso as the most reliable region for non-contact temperature monitoring using the proposed IRT-based method. However, even the torso measurements were subject to potential variations introduced by uncompensated thermal radiation from the incubator and reflections from the IR window. The arms, although having a slightly higher movSD compared to the torso, still provided more reliable peripheral temperature readings with a detection rate of 98.4 %. This offers a viable alternative, especially for early detection of centralization. Conversely, the legs had a significantly lower detection rate of 89.3 %, mainly due to frequent occlusion, making them less suitable for consistent temperature monitoring. Therefore, further investigations of extraction methods are needed to improve measurement reliability in this region.

In summary, future research is essential to validate the extraction method in larger and more diverse populations, to further refine the IRT-based methodology, and to minimize the impact of extraneous factors on measurement accuracy.

## Conclusion

This work successfully demonstrated a novel, non-contact system for the spatial temperature monitoring of premature infants in an incubator using infrared thermography. We developed an integrated pipeline combining a high-accuracy temperature correction algorithm with a robust deep learning model for automated body part segmentation. The system’s ability to provide accurate, spatially resolved temperature data was validated in a clinical setting, showing promising robustness and improvements over traditional wired sensors in terms of patient comfort and nursing workflow.

Despite promising results, limitations such as variations in skin visibility and the complex thermal environment of the incubator must be addressed. Future work should therefore focus on larger clinical trials to validate the system across more diverse populations and on engineering a miniaturized camera system that can be integrated directly into the incubator. Such refinements will further advance this non-invasive technology, with the potential to significantly improve the quality of thermoregulatory care for premature infants.”

## Data Availability

The presented database of infants cannot be made publicly available, due to the declarations in the ethics proposal.
